# Improved intact peptide and protein quantitation by LC‐MS: Battling the deleterious effects of analyte adsorption

**DOI:** 10.1002/ansa.202000102

**Published:** 2020-10-07

**Authors:** EmmaRae L. Murphy, Andrew P. Joy, Rodney J. Ouellette, David A. Barnett

**Affiliations:** ^1^ Atlantic Cancer Research Institute Moncton New Brunswick Canada; ^2^ Department of Chemistry and Biochemistry Mount Allison University Sackville New Brunswick Canada

**Keywords:** adsorption, low‐bind vials, peptide, protein, quantitation

## Abstract

Peptide and protein quantitation by liquid chromatography‐mass spectrometry relies on the assumption of linear signal response with concentration. At low concentrations, analyte adsorption to pipette tips, sample vials and equipment can have significant deleterious effects on signal response. Meanwhile at high concentrations, linearity breaks down due to competitive ionization, signal suppression, and the formation of peptide or protein multimers. These effects result in calibration curves that are more sigmoidal than linear. Linearity at low protein levels for identification and quantitation is of paramount importance in the discovery and validation of biomarker molecules. Herein, we demonstrate the benefits of using commercial low‐bind microcentrifuge tubes and LC vials on the response of a 27‐mer peptide, Vn96, and the intact proteins apomyoglobin and carbonic anhydrase. Linear curves were acquired for Vn96 while apomyoglobin required the addition of intact carbonic anhydrase as an adsorption competitor to achieve linearity. A linear calibration curve for carbonic anhydrase was also acquired by using the polypeptide ubiquitin as an adsorption competitor and internal standard. Linear response was recorded for approximately two orders of magnitude for apomyoglobin and carbonic anhydrase and three orders of magnitude for Vn96 with detection limits ranging from 0.33 to 19 fmol/µL. Finally, we used low‐bind vials for the online enzymatic digestion of apomyoglobin where a high concentration of apomyoglobin acted as an adsorption blocker for the low level trypsin enzyme. Fortunately, the liberated tryptic peptides showed no affinity for the walls of the low‐bind vials. In this study, we take a comprehensive approach to combat analyte adsorption by showing the significance of utilizing low‐bind vials and adsorption competitors to greatly improve upon signal sensitivity at low concentrations of target molecules. The use of these methodologies should improve the low‐level detection of molecules by mass spectrometry.

## 1 INTRODUCTION

Accurate peptide and protein quantitation using mass spectrometry relies on the assumption of a linear relationship between signal intensity and analyte abundance. A typical peptidomics or proteomics experiment is subject to many sources of variability including the sample matrix, solvent composition, salt, detergent and buffer concentration, pH, and vial and pipet tip chemistry and quality.^1^ Regulatory guidelines require bioanalytical methods to be validated in terms of linearity, sensitivity, accuracy, precision, selectivity, reproducibility, robustness, and stability.^2^ Analyte losses due to adsorption in tips, vials, autosampler plumbing, injection valves, and LC columns can introduce bias in both identification and quantification assays.^3^ As analytical technologies become increasingly sensitive, adsorptive losses to vials and equipment components becomes increasingly problematic. Analyte carry‐over from injection to injection can also pose a serious issue to accurate quantitation.

Nonspecific binding of peptides and proteins can essentially occur at the surfaces of all materials used during sample preparation and analysis. Because of the diversity in the physiochemical properties of complex biomolecules, there are currently no universal approaches to minimize the adsorption losses and the phenomenon of analyte carry‐over.

Many studies have encountered peptide signal nonlinearity and have offered a multitude of solutions on how to mitigate this issue. In a study of recovery and repeatability of peptide LC‐MS analysis by van Midwoud et al in 2007,^4^ the authors linked poor study performance to vial quality. They showed significant improvements in analyte sensitivity with the addition of the organic solvent modifier dimethyl sulfoxide (25% DMSO) combined with 5% formic acid. In another study, Stejskal et al^5^ showed that high salt concentrations (ie, 6 M urea and 1 M thiourea) could be used to minimize peptide analyte adsorption but they ultimately settled on the addition of 0.0001% polyethylene glycol to stabilize peptides in solution for several months. A recent 2019 publication by Ranade *et al*. commented on the use of stripping agents to remove tightly adhered milk proteins from sample vials, particularly polypropylene vials.^6^ Interestingly, they found that the metal chelator EDTA enhanced the surface adsorption of milk proteins. In a separate study involving surface interactions with peptides, Goebel‐Stengel et al. evaluated peptide adsorption to several plastic and glassware surfaces^7^ while both Kristensen et al^8^ and Bark et al^9^ also stress the importance of minimizing peptide adsorption on glass and plastics. A study by Pezeshki et al^10^ shows issues of nonlinearity of calibration curves of analytes at high concentrations due to the effects of adsorption, going on to postulate that the effects are worse at low concentrations. They try to address adsorption issues by using adsorption competitors, however their reagents were unable to significantly increase sensitivity. It was found that n‐nonyl‐β‐d‐glucopyranoside gave a slight benefit against the effects of adsorption. All these studies lead to the conclusion that non‐linearity of analyte response with concentration is multi factorial and presents a substantial roadblock to quantitative mass spectrometry. Since adsorption is a concentration dependent effect, it also cannot necessarily be compensated for with the addition of an internal standard. In addition to these above‐mentioned studies, peptide and protein losses can also be caused by improper pH or solvent induced solubility issues.

In this study, we focused on a comprehensive approach to combat adsorption, by investigating three strategies to mitigate analyte losses due to adsorption. The first strategy involved a chemical approach through the addition of an organic modifier (20% acetonitrile),^4^, ^11^, ^12^ and a high concentration (>1.6 M) of sodium chloride to the peptide solution matrix. This strategy showed short‐term success but was ineffective for sample storage times of >2 h (data not shown). The second strategy used in this study involved switching from regular microcentrifuge tubes and LC vials to commercial low‐bind vial options. This strategy proved very effective for a 27‐residue commercial peptide called Vn96 but was ineffective for the quantitative analysis of the intact proteins apomyoglobin and carbonic anhydrase. Our final strategy combined the use of low‐bind vials with the addition of an active‐site blocking agent or adsorption competitor.^6^ In this case, we used the intact protein carbonic anhydrase at a concentration of 10 µg/mL as a blocker for apomyoglobin and the polypeptide ubiquitin at a concentration of 10 µg/mL as a blocker for carbonic anhydrase. While others have used bovine serum albumin or its hydrolyzed form^13^ for this purpose, we chose to use carbonic anhydrase as a competitive adsorption agent, because unlike BSA, its intact form is well resolved from apomyoglobin by gradient reversed‐phase liquid chromatography. We used ubiquitin for calibration of carbonic anhydrase because the two molecules co‐elute and therefore ubiquitin can also be used as an internal standard. We did not take steps in this study to mitigate the expected effects of analyte adsorption to instrument components such as the autosampler, LC columns, ion source or the transfer capillary of the mass spectrometer. Finally we show an application of the low‐bind vials in the online monitoring of the enzymatic digestion of apomyoglobin with trypsin. In this example, the target protein is concentrated enough (10 µg/mL) to protect the low‐level trypsin enzyme (500 ng/mL) from adsorption. Both free and bound apomyoglobin is likely accessible for degradation and the resulting peptides will not adhere to the surfaces of the low‐bind vials.

## 2 METHODS

### 2.1 Vial comparison sample preparation

**FIGURE 1:**
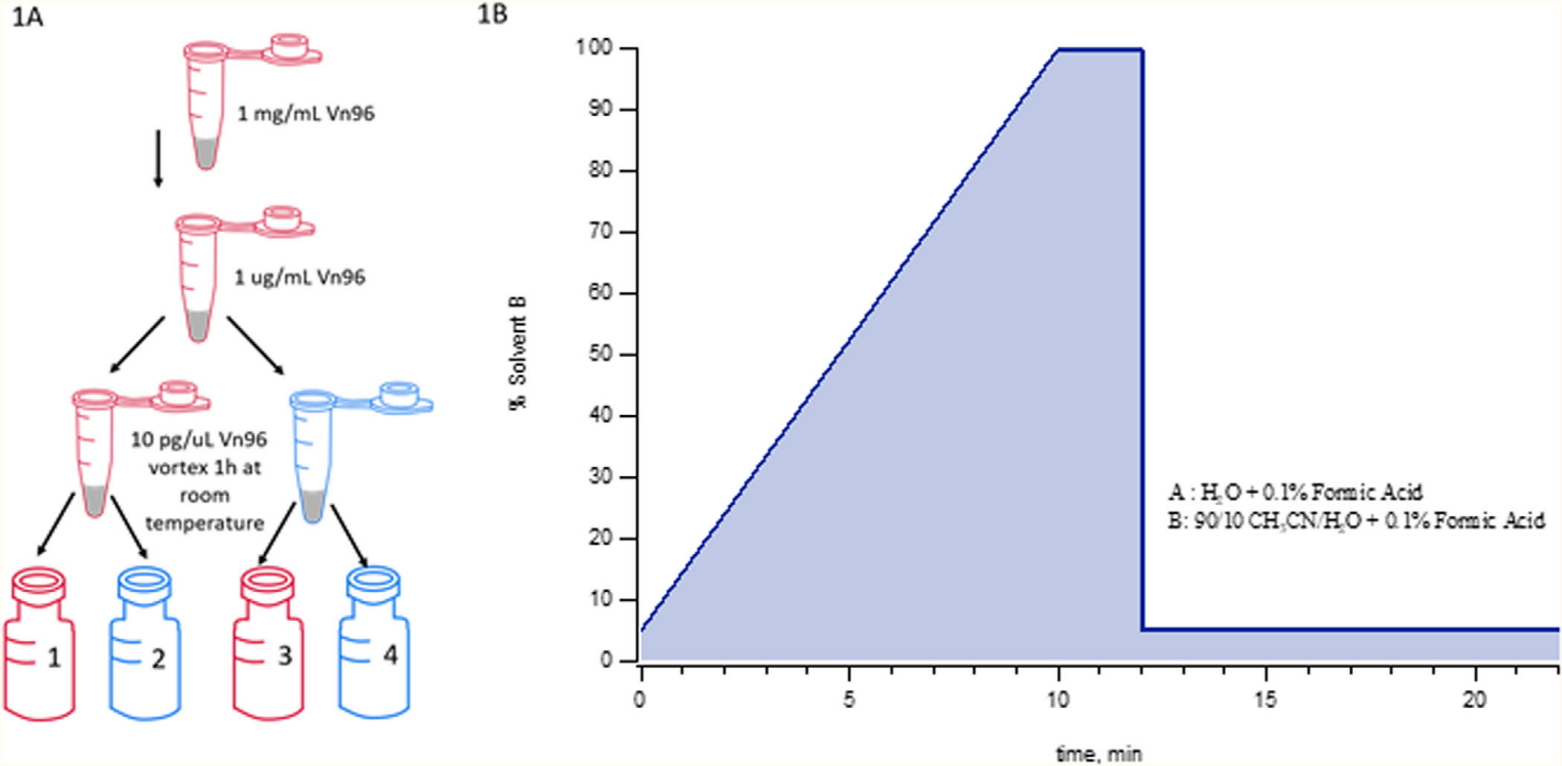
A, Schematic of experimental layout of regular (red) and low‐bind vials (blue) sample preparation. B, The HPLC solvent gradient profile. Upon completion of the gradient, the column was flushed for two minutes and then re‐equilibrated for an additional 10 min. The optimal re‐equilibration time varies with analyte hydrophobicity. In this study, it was found that the necessary re‐equilibration time for apomyoglobin was ∼2 min longer than for the Vn96 peptide

Stock solutions of 1.0 mg/mL Vn96, 10 mg/mL apomyoglobin, 10 mg/mL carbonic anhydrase, and 10 mg/mL ubiquitin were prepared in regular 1.5 mL micro‐centrifuge tubes (Eppendorf, Hamburg, Germany) at the beginning of each experiment and used throughout for the duration of data collection. It is important to note the effect of analyte adsorption at these concentrations is not significant compared to the soluble protein levels. All chemicals used in this study are listed in Table 1. For the initial comparison of vials for quantitation of Vn96, the initial 1.0 mg/mL stock was diluted 1000‐fold to 1.0 µg/mL in a regular 1.5 mL micro‐centrifuge tubes prior to the preparation of the experimental concentration of 10 pg/µL Vn96 in either low‐bind or regular tubes. The dilute sample buffer was composed of 95% acetonitrile, 4.9% water and 0.1% formic acid. The 10 pg/µL standards were vortexed vigorously for one hour at room temperature and then 300 µL was transferred to either a regular LC vial (Canadian Life Science, Peterborough, Canada) or low‐bind LC vial (Waters, Milford, MA) for a total of four samples (Figure 1A) for LC‐MS analysis using the gradient shown in Figure 1B.

**TABLE 1:** Detailed list of chemicals, reagents and solvents used in this study

Abbreviation	CAS #	Accession #	Name	Mol. Wt	Formula	Supplier
ABC	1066‐33‐7		Ammonium Bicarbonate	79.056 Da	NN_4_HCO_3_	Sigma‐Aldrich
H_2_O	7732‐18‐5		Water	18.02 Da	H_2_O	Fisher‐Scientific
ACN	08/05/1975		Acetonitrile	41.05 Da	C_2_H_3_N	Fisher‐Scientific
FA	64‐18‐6		Formic Acid	46.03 Da	C_2_H_2_O_2_	Fisher‐Scientific
Vn96	n/a		Venceremin	2.9 kDa		New England Peptide
UBQ	79596‐22‐4		Ubiquitin	8.56 kDa		Sigma‐Aldrich
TRYP_PIG	9007‐07‐7	P00761	Trypsin‐Gold	∼24.4 kDa		Promega
CAH2_BOVIN	9001‐03‐0	P00921	Carbonic Anhydrase	28.9 kDa		Sigma‐Aldrich
MYG_HORSE	100684‐32‐0	P68082	Apomyogobin	16.9 kDa		Sigma‐Aldrich

### 2.2 Concentration curve and adsorption competitor sample preparation

Six Vn96 standards were prepared ranging in concentration from 0.01 to 15.0 µg/mL in four permutations of regular and low‐bind micro‐centrifuge tubes and LC vials. Six apomyoglobin samples ranging from 0.1 to 30 µg/mL and seven carbonic anhydrase standards ranging from 0.1 to 20 µg/mL were also prepared in one combination of low‐bind micro‐centrifuge tubes and low‐bind LC vials. Following the observation of non‐linear calibration curves for apomyoglobin in low‐bind tubes and vials, new standards were prepared with the addition of 10 µg/mL of intact carbonic anhydrase for apomyoglobin or ubiquitin for carbonic anhydrase as adsorption competitors.

### 2.3 Online tryptic digestion of apomyoglobin

A solution of apomyoglobin was prepared in 50 mM ammonium bicarbonate (ABC) at a concentration of 10 µg/mL and incubated with 0.5 µg/mL of mass spectrometry grade trypsin (Promega, Madison, WA) in a low bind LC vial. The vial was immediately placed in the autosampler at room temperature (∼21°C) upon addition of the trypsin, and the reaction was left to go to completion while the autosampler sampled the vial every 22 min following the gradient outlined in Figure 1B.

### 2.4 Liquid chromatography

A comprehensive list of details of the micro‐scale liquid chromatography methodology are given in Table 2. A Dionex Ultimate 3000 LC (Thermo Fisher Scientific, San Jose, CA) was used for the duration of the study. At the completion of the gradient, as outlined in Table 2, we flushed the column with two column volumes of 100% acetonitrile and then re‐equilibrated for six column volumes of 5% acetonitrile.

**TABLE 2:** Liquid chromatography experimental details

Parameter	Value
Mobile phase flow rate	40 µL/min
Solvent A	H_2_O (0.1% Formic Acid)
Solvent B	90% ACN, 10% H_2_O, 0.1% Formic Acid
Gradient	5‐100% B in 10 minutes
Column flush volume	40 µL/min (2 minutes)
Re‐equilibration volume	40 µL/min (10 minutes)
Column chemistry	C18
Column diameter	1.0 mm
Column length	10 cm
Packing diameter	3 µm
Column temperature	Room Temperature
Injection volume	3 or 5 µL
Column back pressure (5% B)	1625 ‐1675 psi
Mobile phase time (t_m_)	1.6 minutes
Column manufacturer	Avantor® Alltima C18
Column distributor	VWR
Column catalog number	75797‐168

### 2.5 Mass spectrometry

All mass spectrometry detection was performed on a hybrid quadrupole‐Orbitrap tandem mass spectrometer (Q‐Exactive, Thermo‐Fisher Scientific, San Jose, CA), details of the mass spectrometry method are listed in Table 3. The mass spectrometer was calibrated in positive mode every 3 days as per manufacturer recommendations with a commercial calibration mix of caffeine, MRFA and Ultramark (Thermo‐Fisher Scientific)

**TABLE 3:** Quadrupole‐Orbitrap (Q‐Exactive) mass spectrometry experimental details

Parameter	Value
Ion Source	Electrospray, positive mode
Source voltage	3.5 kV
Sheath gas flow rate (arb. Units)	8
Auxiliary gas flow rate (arb. units)	3
Ion transfer capillary temperature	275^o^C
Ion transfer capillary voltage	35
S‐lens voltage	50
Orbitrap resolution	140,000
Orbitrap transient time	512 ms
Maximum ion fill time	500 ms
Mass accuracy	< 10 ppm
Mass range	Variable
Scan functions used	Full‐MS

### 2.6 Data analysis

Raw mass spectrometry data were exported from QualBrowser, Xcalibur version 4.1.5, (Thermo Fisher Scientific, San Jose, CA) into Microsoft Excel version 2010 (Microsoft, Redmond, WA) and then loaded into Igor Pro version 6.37 (WaveMetrics, Lake Oswego, OR) for visualization and fitting of analytical curves.

## 3 RESULTS AND DISCUSSION

### 3.1 Low‐bind micro‐centrifuge tubes and LC vials

**FIGURE 2:**
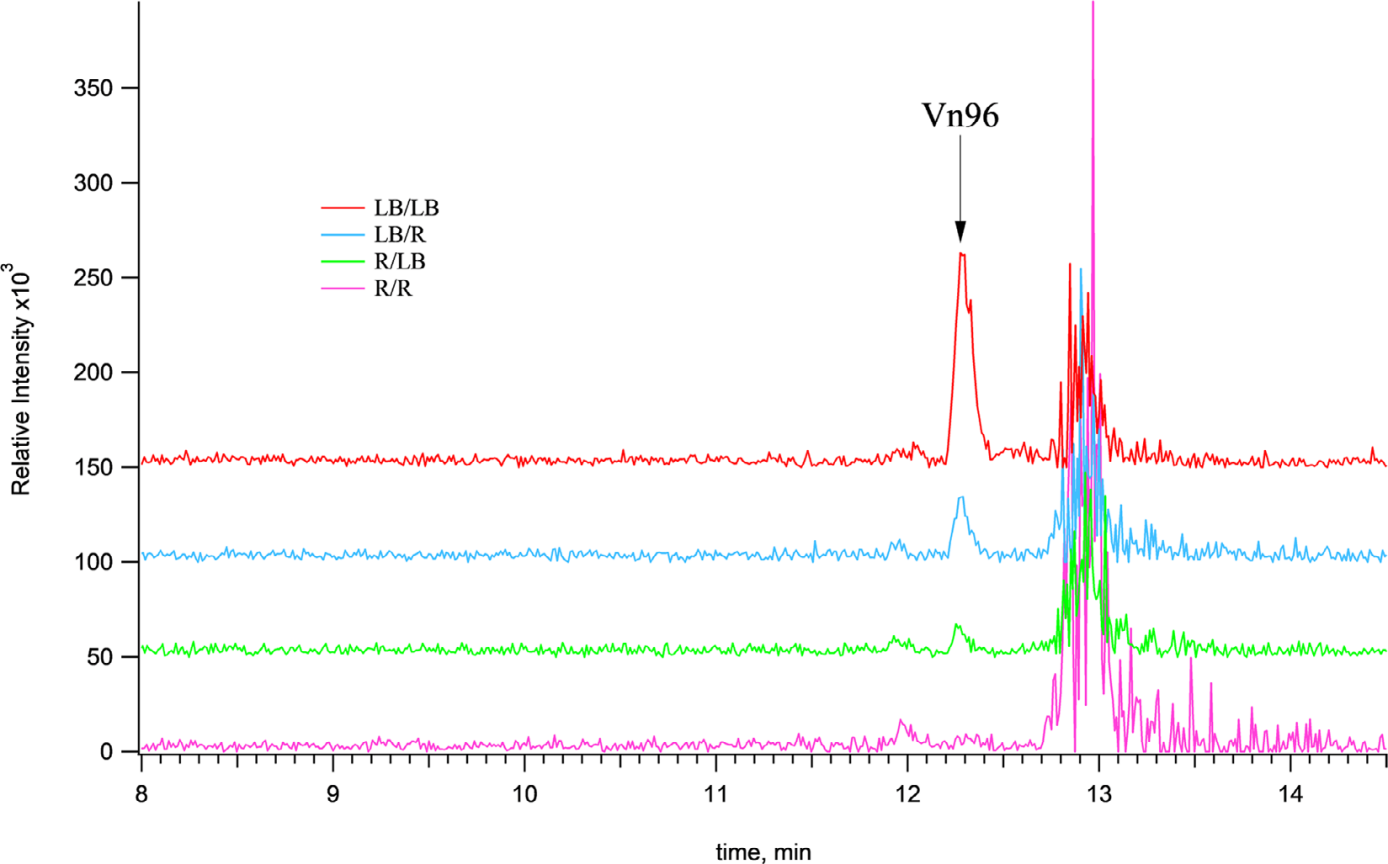
Liquid chromatography‐mass spectrometry of the +4 charge state of a 27‐mer peptide called Vn96 (m/z = 727.4) at a concentration of 10 pg/µL. Peptide solutions were prepared in four different combinations of microcentrifuge tubes and LC vials, namely regular/regular (R/R; pink), regular/low‐bind (R/LB; green), low‐bind/regular (LB/R; blue) and low‐bind/low‐bind (LB/LB; red)

As analytical mass spectrometry becomes increasingly sensitive, scientists are now able to measure smaller amounts of targeted chemicals. This increased sensitivity has exposed other limitations in the analytical protocol. In this paper, we have therefore evaluated the deleterious effect of analyte adsorption to plastics used throughout the sample preparation and processing steps. Vn96 was chosen as a targeted analyte because of its emerging novelty in its ability to isolate extracellular vesicles (EVs).^14^, ^15^ It was important to have an accurate assay for this peptide to determine its method of action and to optimize the EV capture protocol. In previous attempts to develop an assay it was always found that signal for the molecule would suffer significant losses at low concentrations. We eventually attributed this signal loss to the effects of analyte adsorption. We begin by showing a comparison of 4 equimolar preparations of the Vn96 peptide in different combinations of vials. Figure 2 shows chromatograms from four permutations of a 10 pg/µL level of the Vn96 peptide. First, we prepared 1 mL of the 10 pg/µL Vn96 solution from a 1 ng/µL stock into either a regular or low‐bind micro‐centrifuge tube. These two solutions were then vortexed vigorously for 1 h. Three‐hundred microliter aliquots from each of these tubes were then transferred into either regular or low‐bind LC vials and placed in the autosampler. As shown in Figure 2, the expected signal at a retention time of 12.3 minutes for Vn96 is progressively lost when switching between the different permutations of regular and low‐bind tubes and LC vials. It is clear from Figure 2 that all the Vn96 is lost due to adsorption when using regular vials, whereas the best signal for the Vn96 peptide is observed when using all low‐bind microcentrifuge tubes and vials.

### 3.2 Calibration curves for the Vn96 peptide

**FIGURE 3:**
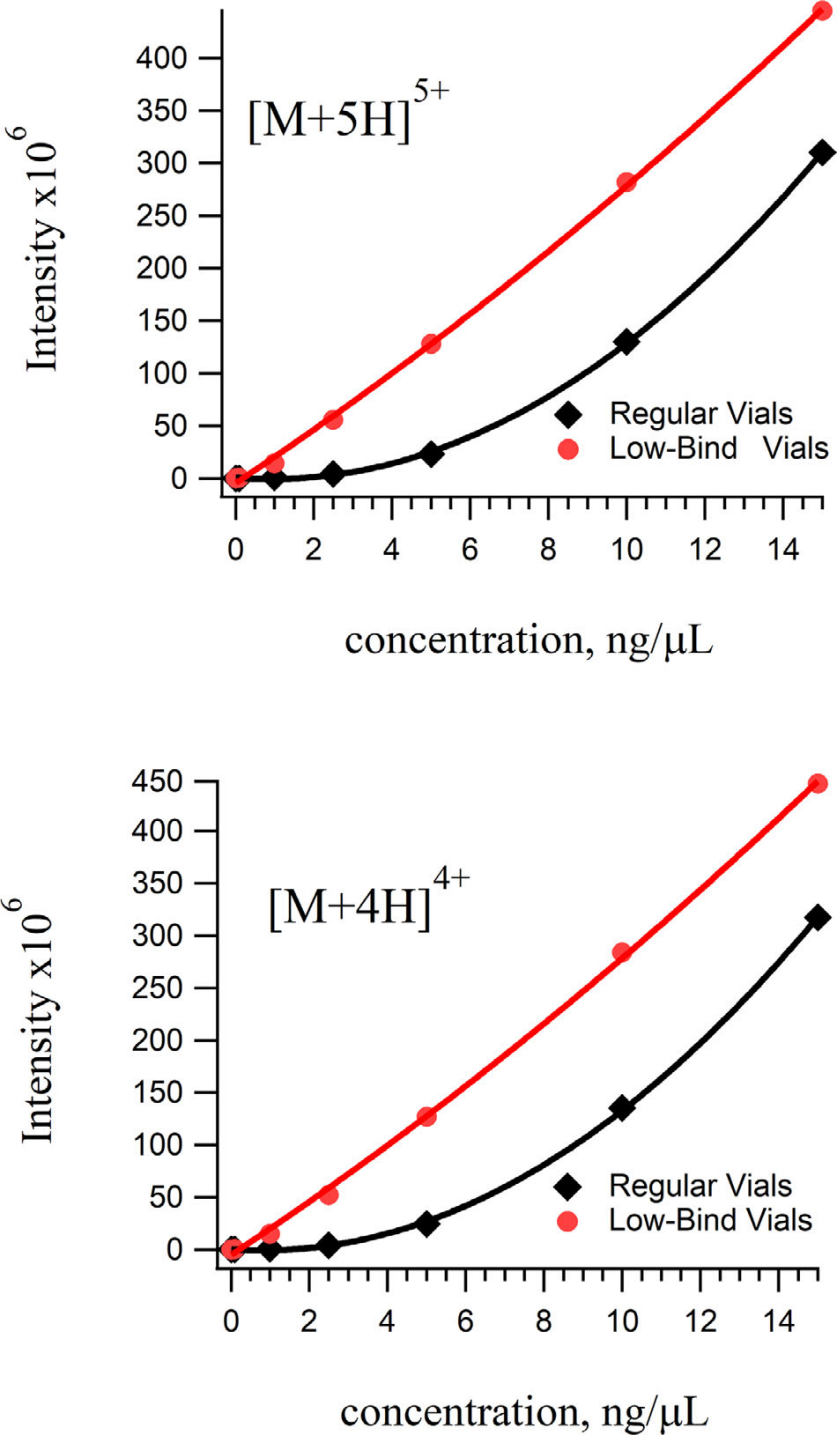
Calibration curves for two charge states, +4 (*m/z* = 727.4) and +5 (*m/z* = 581.9), of Vn96 that was prepared in regular Eppendorf and LC vials compared with the combination of low‐bind/low‐bind vials. The curves are much more linear in the low‐bind vials

**FIGURE 4:**
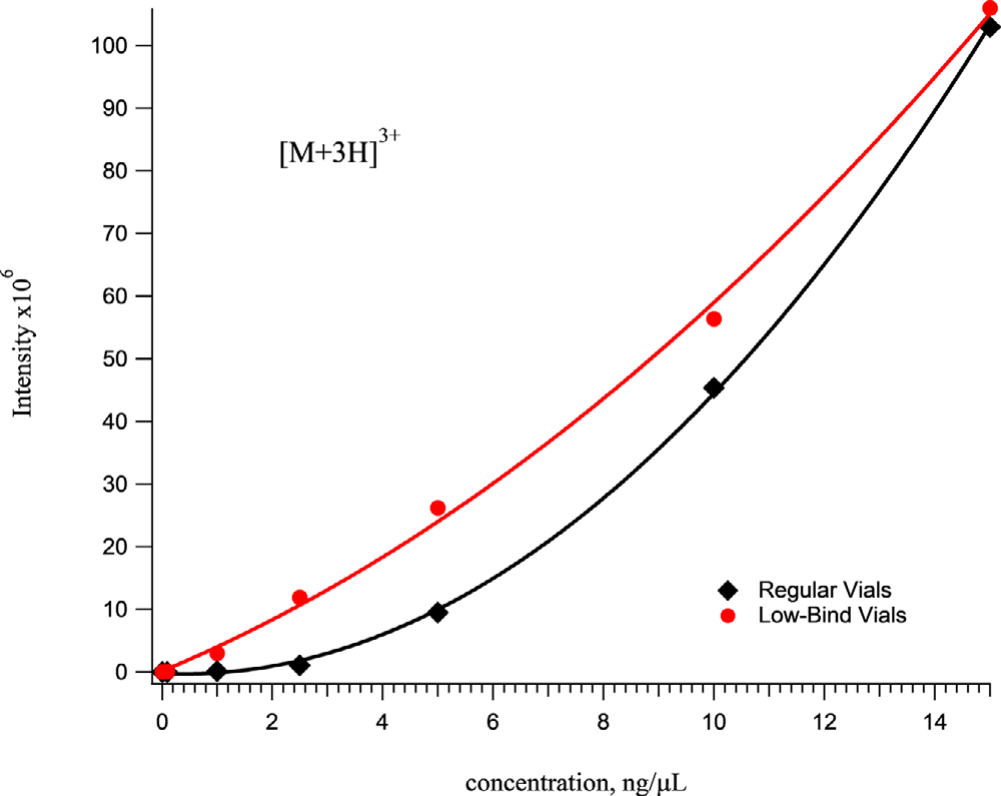
Calibration curves for the +3 charge state (*m/z* = 969.4) of Vn96 prepared in regular versus low‐bind/low‐bind vials. Note that for this charge state, the low‐bind curve is also slightly concave

Figure 3 shows analytical curves for the [M+5H]^5+^ and [M+4H]^4+^ ions of the Vn96 peptide obtained from the injection of samples prepared in either regular or low bind vials. The benefit of using low‐bind vials on signal linearity is quite clear, especially below 5 µg/mL. Figure 4 shows the calibration curve for the [M+3H]^3+^ ion of Vn96. This plot is shown separately because the response in the low‐bind vials is unexpectedly curved. Since the observed charge states in the mass spectrum of Vn96 are independent of the sample vials, this nonlinearity must be due to an artifact of the ionization source. We hypothesize that this non‐linearity is caused by the fact that there are multiple permutations in the Vn96 peptide capable of producing a +3 ion and that ions from each permutation have different ionization efficiencies. By using low bind tubes and vials the sensitivity of the calculated detection limit of intact Vn96 is approximately 0.33 fmol/µL.

### 3.3 Calibration curves for apomyoglobin in low‐bind vials

**FIGURE 5:**
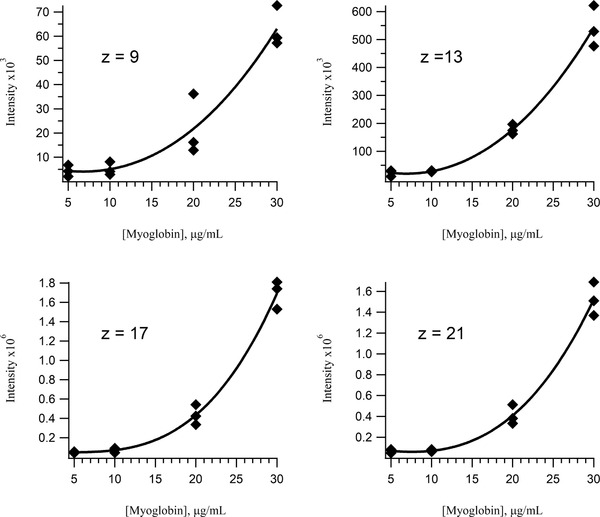
Calibration curves of the intact protein apomyoglobin prepared in low‐bind vials for 4 charge states; + 9 (*m/z* = 1884.2), +13 (*m/z* = 1304.9), +17 (*m/z* = 998.1), and +21 (*m/z* = 808.2). This protein shows significant deleterious effects of analyte adsorption and a very narrow linear dynamic range. Significant ion suppression was observed at a concentration of 40 µg/mL apomyoglobin (not shown)

For our next set of experiments demonstrating calibration curves of intact proteins, we chose apomyoglobin because it is a commonly used standard in the proteomics field that is easily detected in both intact and digested forms. Calibration curves obtained for low‐bind vial preparations of apomyoglobin (Figure 5) show a decrease in sensitivity at low concentrations. This was unexpected given the observed improvement of sensitivity in the curves for Vn96 using the low‐bind vials. This may be because the larger protein has a higher adsorption affinity for the vials or other components in our detection system such as the autosampler, injection valve and/or the ion source of the mass spectrometer, however; the exact reason remains unclear. Despite the uncertainty in the source of the issue, we decided to investigate the effect of adding a blocking agent in the form of another protein molecule. We selected a protein that does not co‐elute with apomyoglobin to avoid isobaric interferences in our mass spectra and therefore used the bovine form of carbonic anhydrase.

### 3.4 Calibration curves for apomyoglobin in low‐bind vials with addition of carbonic anhydrase

**FIGURE 6:**
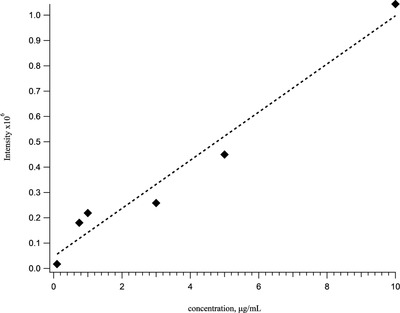
Calibration curves for the +17 (*m/z* = 998.1) charge state of the intact protein apomyoglobin prepared in low‐bind vials in the presence of 10 µg/mL of carbonic anhydrase. To maximize signal‐to‐noise at low levels of apomyoglobin, the mass range was reduced to ±5 Thompson (Th) at 998.1 Th, the maximum ion trap fill time was increased to 1 s and the AGC target value was 3E6

Carbonic anhydrase was added as a competitive adsorption agent because it is a relatively large and hydrophobic protein and it is fully resolved from apomyoglobin by our LC method such that it would not interfere with our ability to quantify low levels of apomyoglobin charge states without requiring fragmentation. A calibration curve for the +17 charge state of apomyoglobin in the presence of 10 µg/mL carbonic anhydrase is shown in Figure 6. Carbonic anhydrase was added to enhance the linearity of the mass spectrometric response. It is unclear whether the carbonic anhydrase is mitigating protein adsorption in the low‐bind vials, the autosampler, injection valve, column, ion source, or mass spectrometer, but it clearly has a positive effect on the response of apomyoglobin. There is also evidence of a “conditioning” effect from adding carbonic anhydrase to the sample vial and column, as the ability to see low levels of apomyoglobin is enhanced even when carbonic anhydrase is no longer in the sample vial (data not shown). In other words, carbonic anhydrase seems to have a lingering effect on the analytical LC‐MS platform. The duration of this lingering effect, however, is currently unclear. The calculated detection limit of intact apomyoglobin using this method is approximately 18 fmol/µL.

### 3.5 Calibration curve for carbonic anhydrase in low‐bind vials with addition of ubiquitin

**FIGURE 7:**
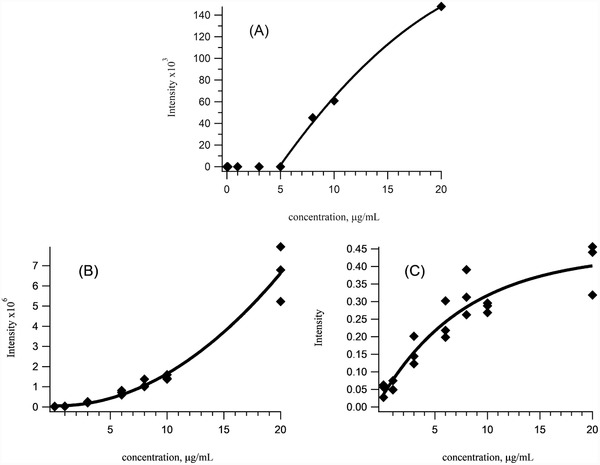
A, Calibration curve for the +18 charge state for carbonic anhydrase (*m/z* = 867.2) in low‐bind vials in the absence of an adsorption blocker. B, Calibration curve for the +18 charge state of carbonic anhydrase in low‐bind vials in the presence of ubiquitin as an adsorption blocker. C, Calibration curve for the +18 charge state of carbonic anhydrase in low‐bind vials in the presence of ubiquitin as an adsorption blocker and internal standard where the curve passes though the origin and is roughly linear up to a concentration of 6 µg/mL

To further evaluate the effects of competitive absorbers on the calibration of intact proteins, we attempted a calibration curve for carbonic anhydrase in low‐bind vials with and without the addition of ubiquitin. Ubiquitin was chosen because it co‐elutes with carbonic anhydrase but fortunately does not interfere with any of the charge states of carbonic anhydrase. The calibration curve from 0.1 to 20 µg/mL in the absence of ubiquitin is shown in Figure 7A. No signal is observed for any of the carbonic anhydrase standards below 8 µg/mL. Figure 7B shows the results of the calibration curve in the presence of 10 µg/mL ubiquitin. While the signal for carbonic anhydrase is curved upward in this plot, if ubiquitin is also used as an internal standard, it is possible to achieve a convex curve that passes through the origin as illustrated in Figure 7C. This final curve is only linear at low concentrations due to the competitive ionization and analyte suppression effects at higher concentrations. The calculated detection limit of intact carbonic anhydrase, using this method, is approximately 19 fmol/µL.

### 3.6 Application of anti‐adsorption strategies to online tryptic digestion

**FIGURE 8:**
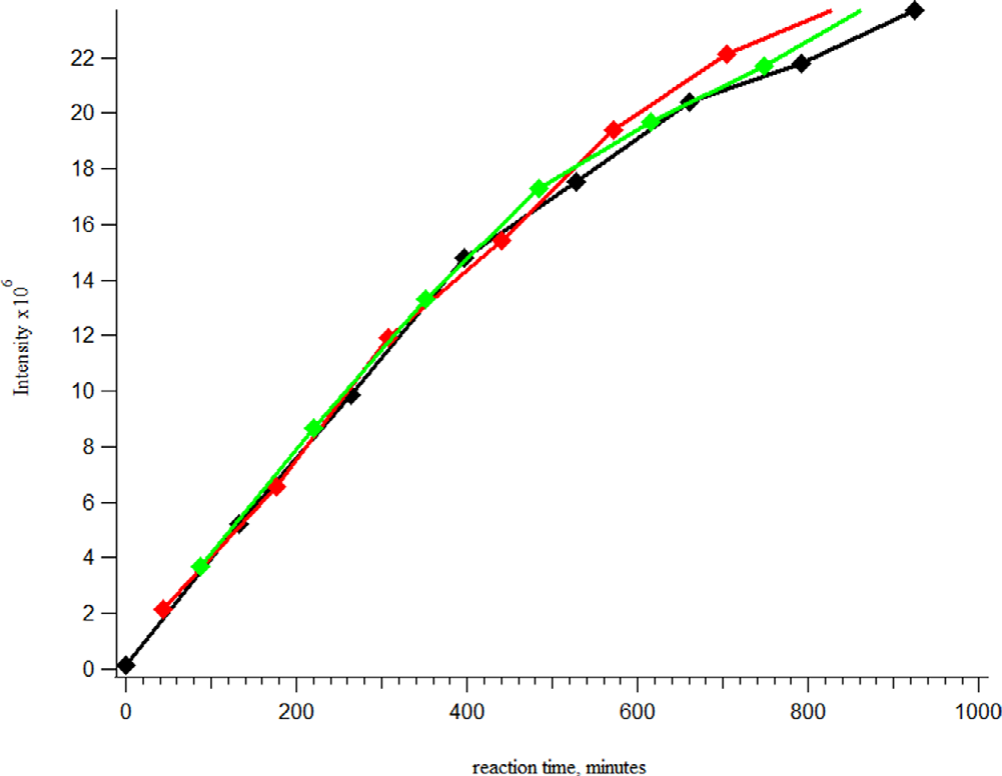
Online tryptic digestion of apomyoglobin showing the appearance of peptide VEADIAGHQEVLIR over time (n = 3). The reaction is monitored to completion and shows a linear response producing an *R* ^2^ value of 0.999

Initially, as observed with detection of intact apomyoglobin in low bind vials in Figure 5, we saw the unexpected loss of sensitivity in low bind vials around 5 µg/mL. We suggested that this was a result of a larger protein having greater affinity than a peptide or smaller protein for adsorption to the walls of a low‐bind vial or instrument components. We hypothesized that if we utilized apomyoglobin at a concentration well above a level where it is completely adsorbed (ie, 10 µg/mL) it could be digested while acting as an adsorber for trypsin at 500 ng/mL. If there is unbound trypsin it should be able to digest both free and bound apomyoglobin. This experiment allows us to observe the digestion of apomyoglobin along with the *in situ* production of several tryptic peptides over time. Enzymatic peptide release is shown to produce a linear response with time until the source protein is depleted. Figure 8 shows a linear response for a tryptic peptide of apomyoglobin with the amino acid sequence VEADIAGHQEVLIR. The reaction was monitored in triplicate from three discrete vials over the same time‐course by injecting from each vial in sequence (ie, vial#1, vial#2 and vial#3) every 22 minutes. The peptides produced by digestion in the low‐bind LC vials show little affinity for the walls of the vials or any of the instrument components.

## 4 CONCLUSIONS

Low‐bind micro‐centrifuge tubes and low‐bind LC vials were used to significantly improve the low‐level quantitative analysis of a model synthetic peptide called Vn96. Trace analysis of apomyoglobin protein required the further addition of an adsorption competitor in the form of an intact protein. While intact albumin protein is often used as pre‐treatment or active site blocker in immunoprecipitation experiments, we did not use it here for the intact analysis of apomyoglobin for two reasons; first, these two proteins co‐elute from the C18 column and the presence of BSA would completely mask the low‐level signal obtained from apomyoglobin. Second, the use of peptides of hydrolysed BSA as competitive adsorbers^13^ was only demonstrated as a solution for reducing adsorption of peptides during vacuum centrifugation. No results were reported for this strategy using intact proteins. We also demonstrate the use of apomyoglobin to protect a low level of trypsin enzyme from adsorption in order to monitor the tryptic digestion of this protein in real time by LC‐MS. Herein we have addressed significant analyte adsorption effects observed for intact proteins in neat or simple solutions. To overcome these negative effects when preparing calibration curves using external standards, we combine the use of low‐bind microcentrifuge tubes and LC vials with the addition of a competitive adsorber in the form another intact protein. From our findings, we propose that the blocking effects of the competitive adsorbers are not unique to the physio‐chemical characteristics of the individual protein, rather it is a concentration dependent effect. We support this argument by using carbonic anhydrase as both a blocker (with apomyoglobin) and an analyte (with ubiquitin as the blocker). When analyzing proteins in samples of much higher complexity (eg, cell lysates, human plasma) we expect that there are already high levels of endogenous blocking molecular species such that the addition of an exogenous protein would not be required. An alternative to adding an adsorber in these circumstances would be the use of the method of standard additions, wherein a recombinant form of the target protein is spiked into the original matrix in at least three discrete levels to make a total of at least four samples. The concentration of the protein is then inferred from these curves as the negative x‐intercept of the curve. While the method of standard additions could be a valuable way to validate quantitative results, it would be impractical for the routine analysis of many samples due to significantly reduced sample throughput. Another potential option for producing relative quantitative results in neat solutions, would be to use an isotopically labeled recombinant form of the target protein at a level where it also serves as an adsorption blocker and could be used as an internal standard. To be a good internal standard, it is imperative that the concentration of the spiked protein lie in a linear range of its response such that small differences in injection volume or instrument response are accurately reflected in its signal.

In this study, we take a comprehensive approach to combat analyte adsorption to demonstrate linear signal for concentration curves over 2‐3 orders of magnitude and detection limits ranging from 0.33 fmol/µL for Vn96 to 18 and 19 fmol/µL for apomyoglobin and carbonic anhydrase respectively. The improved linearity of concentration curves at low concentrations is critical to the successful application of high sensitivity LC‐MS platforms.

## CONFLICT OF INTEREST

The Vn96 synthetic peptide was developed by the Atlantic Cancer Research Institute and commercialized in partnership with New England Peptide (Gardiner, MA).

## References

[1] Kolsrud‐Hustoft H , Malerod H , Ray S , Reubsaet L , Lundanes E , Greibrokk T . A critical review of trypsin digestion for LC‐MS based proteomics. In: Leung H‐C , ed. Integrative Proteomics. London, UK: IntechOpen; 2012.

[2] C. for D. E. and Research and C. for V. Medicine, “Bioanalytical Method Validation Guidance for Industry,” 2018. [Online]. https://www.fda.gov/regulatory-information/search-fda-guidance-documents/bioanalytical-method-validation-guidance-industry. Accessed October 5, 2020.

[3] Rabe M , Verdes D , Seeger S . Understanding protein adsorption phenomena at solid surfaces. Adv Colloid Interface Sci. 2011;162(1‐2):87‐106. 21295764 10.1016/j.cis.2010.12.007

[4] van Midwoud PM , Rieux L , Bischoff R , Verpoorte E , Niederlander HAG . Improvement of recovery and repeatability in liquid chromatography‐mass spectrometry analysis of peptides. J Proteome Res. 2007;6(2):781‐791. 17269734 10.1021/pr0604099

[5] Stejskal K , Potěšil D , Zdráhal Z . Suppression of peptide sample losses in autosampler vials. J Proteome Res. 2013;12(6):3057‐3062. 23590590 10.1021/pr400183v

[6] Ranade AV , Mukhtarov R , Liu KJA , Behrner MA , Sun B . Characterization of sample loss caused by competitive adsorption of proteins in vials using sodium dodecyl sulfate‐polyacrylamide gel electrophoresis. Langmuir. 2019;35(12):4224‐4232. 30813715 10.1021/acs.langmuir.8b04281

[7] Goebel‐Stengel M , Stengel A , Tache Y . The importance of using the optimal plastic and glassware in studies involving peptides. Curr Proteomics. 2011;414(1):38‐46. 10.1016/j.ab.2011.02.009 PMC3290000 21315060

[8] Kristensen K , Henriksen JR , Andresen TL . Adsorption of cationic peptides to solid surfaces of glass and plastic. PLoS One. 2015;10(5):1‐17. 10.1371/journal.pone.0122419 PMC4416745 25932639

[9] Bark SJ , Hook V . Differential recovery of peptides from sample tubes and the reproducibility of quantitative proteomic data. J Proteome Res. 2007;6(11):4511‐4516. 17850064 10.1021/pr070294o

[10] Pezeshki A , Vergote V , Van Dorpe S , et al. Adsorption of peptides at the sample drying step: influence of solvent evaporation technique, vial material and solution additive. J Pharm Biomed Anal. 2009;49(3):607‐612. 19150589 10.1016/j.jpba.2008.12.003

[11] Maes K , Smolders I , Michotte Y , Van Eeckhaut A . Strategies to reduce aspecific adsorption of peptides and proteins in liquid chromatography‐mass spectrometry based bioanalyses: an overview. J Chromatogr A. 2014;1358:1‐13. 25022477 10.1016/j.chroma.2014.06.072

[12] Warwood S , Byron A , Humphries MJ , Knight D . The effect of peptide adsorption on signal linearity and a simple approach to improve reliability of quantification. J Proteomics. 2013;85:160‐164. 23665148 10.1016/j.jprot.2013.04.034 PMC3694305

[13] Verbeke F , Bracke N , Debunne N , Wynendaele E , De Spiegeleer B . LC‐MS compatible antiadsorption diluent for peptide analysis. Anal Chem. 2020;92(2):1712‐1719. 31874035 10.1021/acs.analchem.9b01840

[14] Joy AP , Ayre DC , Chute IC , et al. Proteome profiling of extracellular vesicles captured with the affinity peptide Vn96: comparison of Laemmli and TRIzol© protein‐extraction methods. J Extracell Vesicles. 2018;7(1):1‐12. 10.1080/20013078.2018.1438727 PMC5827780 29511462

[15] Bijnsdorp IV , Maxouri O , Kardar A , et al. Feasibility of urinary extracellular vesicle proteome profiling using a robust and simple, clinically applicable isolation method. J Extracell Vesicles. 2017;6(1):1313091. 28717416 10.1080/20013078.2017.1313091 PMC5505003

